# Changes in serum levels of pain mediators in hemiplegic shoulder pain

**DOI:** 10.1002/brb3.3289

**Published:** 2023-10-20

**Authors:** Mincong Lei, Yidi Wang, Qian Chen, Peng Huang, Yige Li, Yuanyuan Jia, Dianhuai Meng

**Affiliations:** ^1^ Rehabilitation Center The first Affiliated Hospital with Nanjing Medical University Nanjing China; ^2^ Children's Hospital Affiliated to Zhejiang University School of Medicine Hangzhou China; ^3^ Department of Epidemiology School of Public Health, Nanjing Medical University Nanjing China; ^4^ Department of Rehabilitation Medicine Nanjing Qixia District Hospital Nanjing China; ^5^ The Second Affiliated Hospital of Nanjing Medical University Nanjing China

**Keywords:** calcitonin gene‐related peptide, depression, hemiplegic shoulder pain, interleukin‐10, interleukin‐2, poststroke shoulder pain, spasticity, stroke

## Abstract

**Objective:**

To provide a new insight into the diagnosis and treatment of hemiplegic shoulder pain (HSP) by investigating changes in serum pain mediators.

**Design:**

Cross‐sectional study.

**Subjects/patients:**

Shoulder pain group (*n* = 34) and control group (*n* = 21).

**Methods:**

Pain‐free shoulder mobility, anxiety status, depression status, and shoulder pain were measured by passive range of motion (PROM), self‐rating anxiety scale, self‐rating depression scale (SDS), and visual analog scale, respectively. The enzyme‐linked immunosorbent assay was used to test the serum pain mediators, including interleukin (IL)‐1β, IL‐2, IL‐6, IL‐10, nerve growth factor (NGF), tumor necrosis factor‐α (TNF‐α), substance P (SP), calcitonin gene‐related peptide (CGRP), bradykinin (BK), 5‐hydroxytryptamine (5‐HT), prostaglandin E2 (PGE2), and lysophosphatidic acid (LPA).

**Results:**

Shoulder pain group pain‐free PROM significantly lower than control (*p* < .01), and SDS index score of shoulder pain group was significantly higher than control (*p* < .05). The rate of spasticity in the flexor elbow muscles is higher in shoulder pain group (*p* < .01). CGRP, IL‐10, and IL‐2 were significantly upregulated in shoulder pain group compared with control (*p* < .01), whereas NGF, TNF‐α, IL‐6, 5‐HT, PGE2, SP, LPA, BK, and IL‐1β were significantly decreased (*p* < .01).

**Conclusion:**

Patients with HSP have a higher risk of joint mobility disorders and depression; spasticity may be an important factor in the development of shoulder pain; CGRP is thought to be the major pain mediator in HSP, and HSP may not be inflammatory.

## Graphical abstract

The aim of the article was to provide a new insight into the diagnosis and treatment of hemiplegic shoulder pain (HSP) by investigating changes in serum pain mediators. We found that CGRP is thought to be the major pain mediator in HSP, and HSP may not be inflammatory.

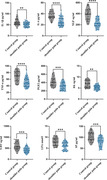

## 1 INTRODUCTION

Hemiplegic shoulder pain (HSP), also known as poststroke shoulder pain, is one of the most common complications after stroke and often affects patients’ quality of life (Li et al., 2022). HSP usually occurs 2 or 3 months after stroke (Anwer & Alghadir, 2020). Incidence rate of HSP varies from 10% to 20%, and its prevalence rate varies from 8% to 47% (Anwer & Alghadir, 2020; Menoux et al., 2019; Nadler et al., 2020; Zhang et al., 2021). The pathogenesis of HSP includes impaired motor control, altered activity of the peripheral or central nervous system, and soft tissue injury, which may occur independently or in concert (Li et al., 2022; Wilson & Chae, 2015). In clinical practice, HSP can be diagnosed by history, musculoskeletal examination, neurological examination, and imaging and can be treated by proper arm handling and positioning, exercise therapy, physical factor therapy, and medication (Dyer et al., 2020; Wilson & Chae, 2015).

Pain mediators are substances involved in the perception of pain that are released after peripheral stimulation and central interpretation and include both pain‐inducing and analgesic substances (Widgerow & Kalaria, 2012). There are no previous studies measuring pain mediators in HSP. In order to gain an understanding of the pain mediators that may be associated with HSP, we reviewed studies of shoulder pain or joint disease.

A review of complex regional pain syndromes (CRPS) suggests that mediators including substance P (SP), calcitonin gene‐related peptide (CGRP), bradykinin (BK), interleukin (IL)‐1β, IL‐2, IL‐6, IL‐10, and tumor necrosis factor‐α (TNF‐α) may be altered during the acute period causing shoulder pain and other symptoms (Bruehl, 2015). In rotator cuff disease, Izumi et al. (2021) found that SP and nerve growth factor (NGF) increased in the degenerative biceps long head tendon may be responsible for the pain. Shih et al. (2018) found a positive correlation between IL‐1β levels in the shoulder joint fluid and pain in chronic rotator cuff tears. In addition, Nagura et al. (2019) found that IL‐1β was regulated to produce NGF and cyclooxygenase‐2 (COX‐2) in rotator cuff patients, COX‐2 further produced prostaglandin E2 (PGE2), leading to shoulder pain. Another study found that 5‐hydroxytryptamine (5‐HT) in synovial fluid of the temporomandibular joint contributes to temporomandibular joint pain (Oliveira‐Fusaro et al., 2012). McDougall and Reid (2022) found that lysophosphatidic acid (LPA) could cause joint degeneration and neuropathic pain in rats. Different mediators have been implicated in neuropathic, inflammatory, and nociceptive pain (Scholz & Woolf, 2002). In inflammatory pain, immune cells and damaged tissue release mediators such as IL‐1β, IL‐2, IL‐6, IL‐10, NGF, TNF‐α, BK, 5‐HT, and PGE2, resulting in an “inflammatory soup” (Scholz & Woolf, 2002). In conclusion, the abovementioned mediators IL‐1β, IL‐2, IL‐6, IL‐10, NGF, TNF‐α, SP, CGRP, BK, 5‐HT, PGE2, and LPA are associated with shoulder pain or joint dysfunction.

As is well known, the identification of pain mediators in pain‐related diseases is a potential avenue for the development of novel targeted drugs, more effective diagnostics, and the exploitation of mechanism‐based treatments (Bannister et al., 2020; Bhansali et al., 2021; Mangnus et al., 2022; Shraim et al., 2022). However, it is currently unclear which pain mediators are involved in HSP and which types of pain are present in HSP. This study was a preliminary investigation to determine changes in serum mediators in HSP and the relationship between HSP and inflammatory pain.

## 2 METHODS

### 2.1 Study design

This is a cross‐sectional study. This study was approved by the Ethics Committee (Ethics Approval No. 2022‐SR‐015) and completed clinical trial registration in the Clinical Trial Registry (Registration No. ChiCTR2100054075). All enrolled patients were well informed about the procedures and objectives before signing an informed consent form.

### 2.2 Participant

Patients in this study were recruited from hospitals between December 2021 and June 2022. Based on previous studies measuring pain mediators in CRPS (Alexander et al., 2005; Lenz et al., 2013), a total of 55 patients were enrolled in this study, including the shoulder pain group (*n* = 31) and the control group (*n* = 24).

Inclusion criteria for the shoulder pain group were as follows: (i) first unilateral cerebral hemorrhage or infarction confirmed by head MRI or CT; (ii) age 18–85 years; (iii) mini‐mental state examination ≥25; (iv) basic vital signs are stable; (v) poststroke patients with shoulder pain and visual analog scale (VAS) ≥ 3. Exclusion criteria for the shoulder pain group: (i) shoulder pain caused by other diseases; (ii) pain in other parts of the body; (iii) history of shoulder injury or pain; (iv) have a serious disease; (v) have other diseases that affect mediators, such as inflammatory diseases and rheumatic immune diseases; (vi) oral analgesics during the first 2 weeks of enrollment; and (vii) pregnant or preparing for pregnancy. According to the above criteria, VAS = 0 was included in control group. The inclusion criteria of the control group were almost the same as those of the shoulder pain group, except “(v) poststroke patients without any pain and VAS = 0.”

### 2.3 Serum collection and pain mediator test

In all patients, 5 mL of hemiplegic anterior cubital vein blood was drawn by experienced nurses at 6 am. The blood was naturally coagulated at room temperature for 10–20 min and centrifuged for 20 min at 2000 RPM. Collect the supernatant after centrifugation and store it in the refrigerator at −80°C until the test. If there is precipitation, centrifuge it again.

CGRP, NGF, TNF, 5‐HT, PGE2, SP, IL‐1β, IL‐2, IL‐6, IL‐10, LPA, and BK in serum were determined by enzyme linked immunosorbent assay (ELISA) (models and specifications show in Appendix 1). In this paper, NGF, 5‐HT, and BK were measured in ng/mL, LPA in μmol/L, and other pain mediators in pg/mL. Each pain mediator was measured three times for each sample using two 96‐well plates. The standard curve range and sensitivity of this method were CGRP 3.75–120 and 0.1 pg/mL, NGF 15.625–500 and 1.0 ng/mL, TNF‐α 2.5–80 and 0.1 pg/ mL, 5‐HT 25–800 and 1.0 ng/mL, PGE2 20–640 and 1.0 pg/mL, SP 3.75–120 and 0.1 pg/mL, IL‐1β 2.5–80 and 0.1 pg/mL, IL‐2 25–800 and 1.0 pg/mL, IL‐6 1.5–48 and 0.1 pg/mL, IL‐10 25–800 and 1.0 pg/mL, LPA 0.25–8 and 0.1 μmol/L, and BK 0.25–8 and 0.1 ng/mL. The optical density of all samples is higher than that of the blank and falls within the linear range of the standard curve.

### 2.4 Outcome measures

Demographic and clinical data were used to collect the appropriate information for the study. The primary outcome was shoulder pain rating using VAS. Secondary outcomes were as follows: (i) neurological function after stroke as measured by the NIHSS score; (ii) pain‐free passive range of motion (PROM) of hemiplegic shoulder abduction and external rotation; (iii) activity of daily living as measured by the Barthel Index (BI); (iv) anxiety status as assessed by the self‐rating anxiety scale (SAS); and (v) depression status as assessed by the self‐rating depression scale (SDS).

### 2.5 Data analysis

The Shapiro–Wilk method was used to test the type of data distribution for all data. Data were under a normal distribution, such as BMI in baseline, which were described as mean (SD) and compared between groups using the *t*‐test. Data with skewed distribution, such as other baseline data except BMI, primary and secondary data, and pain mediator levels, were described by median and interquartile range (M, IQR), and compared between groups using the Mann–Whitney *U* test and the Kruskal–Wallis *H* test. Count data were described as frequency and percentage *n* (%), and the *χ^2^* test was used for comparison between groups. A bilateral *p* < .05 was considered statistically significant. SPSS 20.0 (IBM) was used for statistical analysis, and GraphPad Prism 9.0 was used for graphing.

## 3 RESULTS

### 3.1 Patient characteristics

**FIGURE 1:**
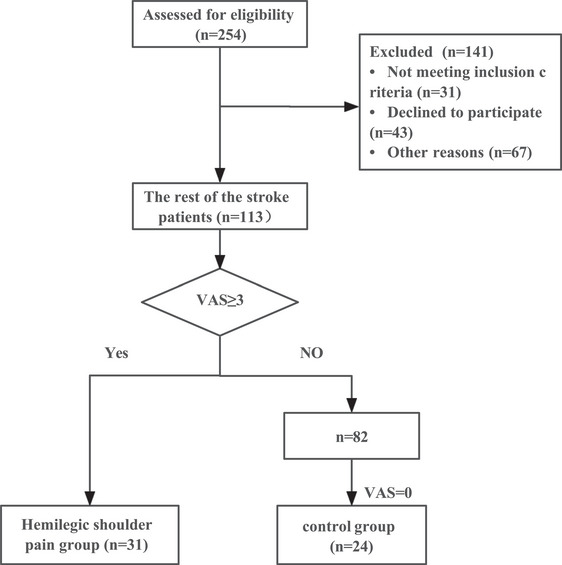
Flowchart of patients screening.

The flowchart is shown in Figure 1. Baseline information is presented in Table 1. The stroke duration of shoulder pain group [70.00 (42.00) d] was significantly higher than that of control group [37.00 (43.00) d] (*p* < .05). The rate of spasticity in the flexor elbow muscles is higher in shoulder pain group (64.52%) than control (29.17%) (*p* < .05). There was no significant difference in other baseline information between two groups (*p* > .05).

**TABLE 1:** Baseline.

		Shoulder pain group (*n* = 31)		Control group (*n* = 24)		*t*/*Z*/*χ* ^2^	*p*
gender, *n*, %							
	Male	20 (64.52)		18 (75.00)		.70	.404
	Female	11 (35.48)		6 (25.00)			
Age, year, median (IQR)		64.00 (15.00)		59.00 (14.00)		1.22	.221
BMI, (kg/m^2^), mean (SD)		24.52 ± 3.22		23.67 ± 3.44		.95	.349
Stroke duration, d, median (IQR)		70.00 (42.00)		37.00 (43.00)		2.99	.003**
Stroke type, *n* %						.33	.568
	Hemorrhage	14 (45.16)		9 (37.50)			
	Infarction	17 (54.84)		15 (62.50)			
Hemiplegic side, *n*, %							
	Left	16 (51.61)		14 (58.33)		.25	.620
	Right	15 (48.39)		10 (41.67)			
Hypertension, *n*, %							
	No	7 (22.58)		8 (33.33)		.79	.375
	Yes	24 (77.42)		16 (66.67)			
Diabetes, *n*, %							
	No	19 (61.29)		16 (66.67)		.17	.681
	Yes	12 (38.71)		8 (33.33)			
MAS (Shoulder adduction), *n*, %							
No spasticity	0	26 (83.87)		22 (91.67)			
	1	3		2		.74	.651
Have spasticity	1+	1	5 (16.13)	0	2 (8.33)		
	2	1		0			
MAS (elbow flexion), *n*, %							
No spasticity	0	11 (35.48)		17 (70.83)			
	1	12		5		6.76	.009**
Have spasticity	1+	7	20 (64.52)	1	7 (29.17)		
	2	1		1			

Abbreviations: BMI, body mass index; IQR, interquartile range; MAS, modified ashworth scale; SD, standard deviation. *p < .05.

**p < .01.

To learn whether the difference in stroke duration affects the comparative result of the levels of pain mediators in two groups, we divided 24 patients of control group into three, based on “0–30 days (10 patients),” “31–60 days (8 patients),” and “>60 days (6 patients).” There was no significant difference in the levels of 12 pain mediators among the three groups, indicating that serum levels of pain mediators do not change with stroke duration in the control group. Thus, duration difference may have little influence on the level of pain mediator comparison (Table 2).

**TABLE 2:** Comparison of pain mediators in the control group at different times.

	Stroke duration of control group			*H*	*p*
	0–30 days (*n* = 10)	31–60 days (*n* = 8)	>60 days (*n* = 6)		
NGF (ng/mL)	384.92 (201.66)	421.65 (196.75)	393.86 (137.28)	0.47	.789
SP (pg/mL)	92.19 (27.71)	103.72 (41.78)	114.21 (25.14)	0.82	.663
PGE2 (pg/mL)	370.71 (136.23)	462.29 (249.19)	376.57 (297.03)	2.17	.339
TNF (pg/mL)	74.40 (23.55)	55.53 (34.73)	71.56 (26.01)	1.45	.486
IL‐1β (pg/mL)	65.05 (16.04)	54.35 (7.41)	57.48 (31.73)	2.37	.305
IL‐2 (pg/mL)	444.30 (216.26)	583.54 (203.52)	680.32 (84.61)	5.59	.061
IL‐6 (pg/mL)	34.30 (9.33)	40.19 (10.08)	34.09 (19.80)	0.43	.806
IL‐10 (pg/mL)	323.64 (181.75)	377.71 (235.81)	296.48 (211.77)	0.40	.819
CGRP (pg/mL)	59.57 (19.29)	58.29 (25.43)	45.71 (37.94)	1.89	.390
BK (ng/mL)	6.00 (2.88)	5.18 (3.68)	7.84 (1.54)	3.33	.190
5‐HT (ng/mL)	638.65 (349.14)	622.12 (376.27)	554.81 (198.87)	1.14	.565
LPA (μmol/L)	7.62 (4.53)	8.84 (3.55)	7.78 (2.36)	0.47	.790

Abbreviations: BK, bradykinin; CGRP, calcitonin gene‐related peptide; 5‐HT, 5‐hydroxytryptamine; IL‐10, interleukins‐10; IL‐1β, interleukins‐1β; IL‐2, interleukins‐2; IL‐6, interleukins‐6; LPA, lysophosphatidic acid; NGF, nerve growth factor; PGE2, prostaglandin E2; SP, substance P; TNF‐α, tumor necrosis factor‐α.

### 3.2 Primary and secondary assessment outcome

The results are shown in Table 3. VAS were [M (IQR), 4.50 (2.00)] in shoulder pain group and [M (IQR), 0.00 (0.00)] in control group. Secondary outcome measures that showed statistically significant differences (*p* < .05) included the shoulder pain‐free PROM for abduction [shoulder pain group: 86.00 (22.50)° vs. control group: 86.00 (22.50)°] and external rotation [shoulder pain group: 0.00 (0.00)° vs. control group: 90.00 (0.00)°] and the index score of SDS [shoulder pain group: 33.13 (9.38) vs. control group: 40.00 (13.75)]. In contrast, NIHSS, BI, and SAS index score were not significantly different between the two groups (*p* > .05), indicating that they were similar in terms of neurological function, ability of daily living, and anxiety levels.

**TABLE 3:** Primary and secondary assessment outcome.

		Shoulder pain group (*n* = 31)	Control group (*n* = 24)	*Z*	*p*
VAS		4.50 (2.00)	0.00 (0.00)	–	–
NIHSS		6.00 (6.00)	3.50 (6.25)	1.08	.297
Shoulder pain‐free PROM (°)	Abduction	86.00 (22.50)	180.00 (0.00)	6.20	<.001***
	External rotation	0.00 (0.00)	90.00 (0.00)	5.92	<.001***
ADL (BI)		50.00 (30.00)	50.00 (45.00)	0.23	.818
Index score of SAS		37.50 (12.50)	33.13 (10.00)	1.70	.089
Index score of SDS		40.00 (13.75)	33.13 (9.38)	2.30	.022*

Abbreviations: ADL, activity of daily life; BI, barthel index; NIHSS, national institute of health stroke scale; PROM, passive range of motion; SAS, self‐rating anxiety scale; SDS, self‐rating depression scale; VAS, visual analogue scale.

*p < .05.

***p < .001.

### 3.3 Test results for pain mediators

**FIGURE 2:**
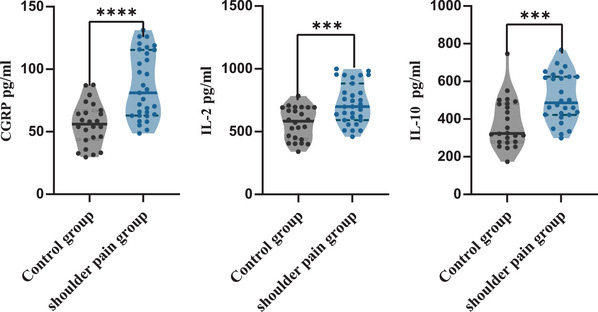
The elevated pain mediators in shoulder pain group. (a) CGRP, calcitonin gene‐related peptide; IL‐2, interleukins‐2; IL‐10, interleukins‐10. (b) Significantly upregulated pain mediators in hemiplegic shoulder pain (HSP) (Mann–Whitney *U* test); (c) The violin plot above shows median, interquartile range, and single data, ***p < .001, ****p < .0001.

**FIGURE 3:**
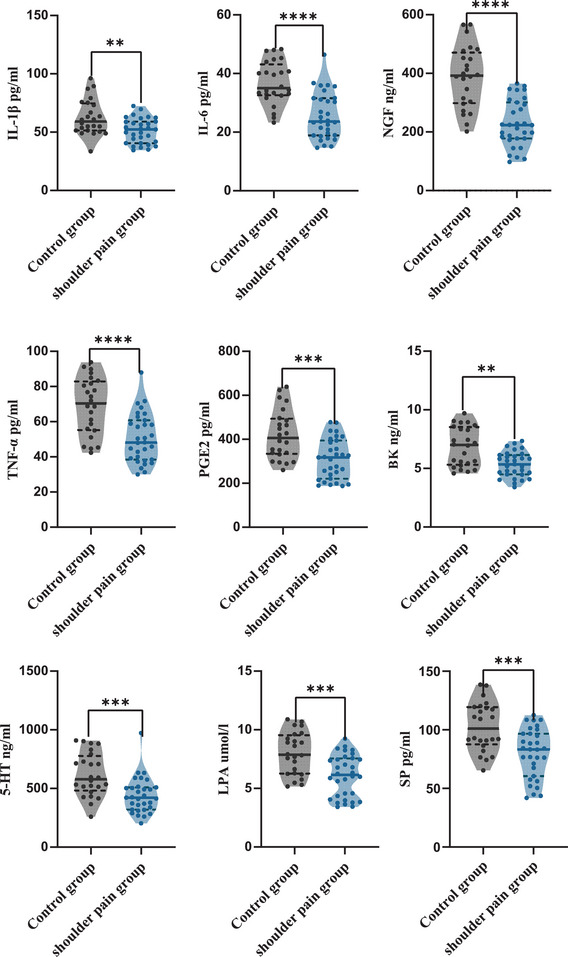
The decreased pain mediators in shoulder pain group (a). 5‐HT, 5‐hydroxytryptamine; BK, bradykinin; IL‐1β, interleukins‐1β; IL‐6, interleukins‐6; LPA, lysophosphatidic acid; NGF, nerve growth factor; PGE2, prostaglandin E2; SP, substance P; TNF‐α, tumor necrosis factor‐α. (b) Significantly downregulated pain mediators in hemiplegic shoulder pain (HSP) (Mann–Whitney *U* test). (c) The violin plot above shows median, interquartile range and single data. ***p < .001, ****p < .0001.

The levels of 12 pain mediators were significantly different between the two groups (*p* < .01) (Figures 2 and 3). Serum levels of CGRP [M (IQR): 80.89 (52.34) pg/mL], IL‐2 [M (IQR): 699.74 (290.58) pg/mL], and IL‐10 [M (IQR): 497.40 (201.68) pg/mL] levels in shoulder pain group were significantly upregulated compared to CGRP [M (IQR): 56.04 (22.81) pg/mL], IL‐2 [M (IQR): 583.54 (252.57) pg/mL], and IL‐10 [M (IQR): 323.64 (202.99) pg/mL] levels in control (*p* < .05). The levels of NGF, TNF‐α, IL‐6, 5‐HT, PGE2, SP, LPA, BK, and IL‐1β were significantly downregulated (*p* < .01).

## 4 DISCUSSION

The results of this study showed that pain‐free PROM in shoulder pain group was significantly decreased and the index score of SDS was significantly increased than control. This suggests that HSP may affect the pain‐free mobility of the shoulder joint and HSP patients were more likely to have depressive tendencies than control. Decreased pain‐free PROM in the shoulder may arise from various factors such as insufficient active and passive motion, tissue adhesions, spasticity in shoulder muscles, soft tissue lesions, and altered peripheral and central nervous activity among others (Wilson & Chae, 2015). Early attention to shoulder range of motion should be recommended. In addition to conventional rehabilitation management methods to improve shoulder range of motion, injection therapy has been shown to be effective (Wilson & Chae, 2015). Examples of these therapies include intramuscular botulinum toxin injections to reduce spasticity, intra‐articular, or bursal corticosteroid injections for rotator cuff pathology, and suprascapular nerve blocks to reduce shoulder pain caused by capsule and ligament damage (Chiu et al., 2021; Wilson & Chae, 2015; Wong, 2023).

The results of this study showed that a greater number of individuals in shoulder pain group suffered from spasticity in the flex elbow muscles. This indicates that spasticity might be a relevant factor in the occurrence of shoulder pain. Jia et al. (2023). found that the stiffness of teres major was associated with the level of pain and decreased mobility in HSP. In contrast, Wong (2023). found no correlation between pain and spasticity in with spastic cerebral palsy. Therefore, further confirmation is needed to determine whether spasticity causes pain. There are many ways to reduce spasticity, such as extracorporeal shock wave therapy, intrathecal baclofen injection, and intramuscular botulinum toxin injection (Creamer et al., 2018; Kaku & Simpson, 2016; Wissel et al., 2016).

The results of this study showed that the index score of SDS was significantly higher than control. This suggests that HSP patients were more likely to have depressive tendencies. At present, studies of the relationship between non‐HSP and emotional state are more than that of HSP. Cho et al. (2013) observed shoulder pain patients lasting more than 3 months and found them more prone to anxiety, depression, and sleep disturbances than the healthy. Another systematic review showed that anxiety and depressive symptoms increase patients' perception of pain. In addition, the fear of pain and somatic catastrophizing further upper extremity function on the affected side (Brindisino et al., 2022). The result suggests that there is an association between HSP and depressive states. Thus, shoulder pain patients should not only focus on the pain symptom but also pay attention to the depression status.

The results of this study showed that serum IL‐2, L‐10, and CGRP were higher in shoulder pain group than in control. IL‐1β, IL‐6, NGF, TNF‐α, SP, BK, 5‐HT, PGE2, and LPA in shoulder pain group were lower than those in control group.

IL‐2 and IL‐10 are known to have analgesic effects and can be used for diagnosis and treatment. A study of exercise‐induced musculoskeletal pain found that plasma IL‐10 was elevated 48 h after injury, which may be associated with inflammation and pain (Hedderson et al., 2020). Yao et al. (2002) has found that the direct intrathecal injection of the IL‐2 gene has an antinociceptive effect, which may be a way to relieve neuropathic pain. Frangiamore et al. (2017). found that the detection of IL‐2 and IL‐10 levels in synovial fluid during shoulder replacement surgery is a promising method for diagnosing and predicting postoperative peri‐shoulder infection. In this study, IL‐2 and IL‐10 may also play an anti‐inflammatory and analgesic role in HSP. At present, no further studies have been conducted on the changes in IL‐2 and IL‐10 in HSP, which require further investigation. Whether IL‐2 and IL‐10 can be used for diagnosis or pain relief also needs further exploration.

IL‐1β, IL‐6, NGF, TNF‐α, SP, BK, 5‐HT, PGE2, and LPA function at different stages of the pain pathway, leading to inflammatory pain. Yuan et al. (2020) found that the upregulation of the NGF gene can perpetuate chronic inflammatory pain. Choi and Hwang (2018) proposed that BK causes inflammatory pain by increasing the excitation of nociceptive neurons. Nakajima et al. (2009) found that 5‐HT binding to the 5‐HT2A and 5‐HT2C receptors caused acute inflammatory pain in the rat hind paw and that local antagonism of 5‐HT could relieve pain. LPA binding to the LPA1 receptor in the central nervous system could cause oral and facial inflammatory pain (Srikanth et al., 2018). SP also plays an essential role in oral and facial inflammatory pain (Zhang et al., 2019). IL‐6 and TNF‐α induce central nervous system glial cells to release proinflammatory cytokines TNF‐α, IL‐1β, and IL‐6 (Di Maio et al., 2023). TNF‐α, IL‐1β, and IL‐6 can further induce the production of COX‐2 and increase PGE2 (Zhao et al., 2022). PGE2 is an important mediator which critical to inflammation by causing over‐excitation of sensory neurons leading to hypersensitivity to pain (Di Maio et al., 2023). The aforementioned pain mediators are involved in inflammatory pain, but in this study, the levels of them are not elevated in shoulder pain group, suggesting that HSP may not be inflammatory pain.

CGRP is the only one elevated pain inducing mediator in this study. So, it may be the major mediator in HSP. Research on CGRP has rarely focused on shoulder pain. Alpar et al. (2002) found that serum CGRP was significantly higher in patients with chronic shoulder and neck pain caused by whiplash than in healthy subjects. Onuoha and Alpar (1999) found that patients with soft tissue injuries caused by sprains, ligament instability, and muscle tears have elevated plasma CGRP. Soft tissue injury was one of the mechanisms leading to HSP (Wilson & Chae, 2015). The elevated serum CGRP in HSP considered to be released by injured soft tissue, but this needs more research to confirm. As for treatment, there are some drugs could block the pathway of CGRP, but they were primarily focused on migraine (Rees et al., 2022). Whether they can be used in HSP patient needs more research.

The limitation of the study was that the stroke duration between the two groups was different, and shoulder pain group was higher. Possibly because HSP usually occurs 2–3 months after stroke (Anwer & Alghadir, 2020), participants in control group does not occur pain. We divided control groups into three groups with different duration and found that pain mediators do not change in control, indicating that stroke duration difference does not affect pain mediators’ comparison between shoulder pain group and control group. In the future, the mechanism of CGRP, IL‐2, and IL‐10 in HSP may be explored. Based on this, more mechanism‐based drugs can be developed.

In conclusion, this study found that serum CGRP, IL‐2, and IL‐10 were increased in HSP patients, indicating that CGRP may be the major pain‐inducing mediator, and IL‐2, IL‐10 may contribute to anti‐inflammatory and analgesic effects. Inflammation‐related mediators IL‐1β, IL‐6, NGF, TNF‐α, SP, BK, 5‐HT, PGE2, and LPA were decreased, and HSP may not be inflammatory pain. In addition, HSP patients are more prone to joint mobility problems and depression than non‐HSP stroke patients. Spasticity may play a role in causing shoulder pain, and spasticity should be considered when treating shoulder pain. Furthermore, this study provides a new perspective to diagnose or treat HSP from pain mediators.

## AUTHOR CONTRIBUTIONS

**Mincong Lei**: Conceptualization; methodology; writing—original draft. **Yidi Wang**: Formal analysis; resources. **Qian Chen**: Investigation; resources. **Peng Huang**: Methodology. **Yige Li**: Validation; data curation. **Yuanyuan Jia**: Conceptualization; data curation. **Dianhuai Meng**: Conceptualization; methodology; writing—review and editing; supervision.

## CONFLICT OF INTEREST STATEMENT

The authors declare no conflicts of interest.

### PEER REVIEW

The peer review history for this article is available at https://publons.com/publon/10.1002/brb3.3289.

## Data Availability

The data are not publicly available due to privacy or ethical restrictions; research data are not shared.

## APPENDIX 1 Name, model, and specification of the Elisa.

### 1.1

**Table:**

Model	Name	Specification
MM‐1545H1	Human nerve growth factor (NGF) ELISA Kit	96T
MM‐1506H1	Human substance P (SP) ELISA Kit	96T
MM‐0162H1	Human prostaglandin E2(PGE2) ELISA Kit	96T
MM‐0122H1	Human tumor necrosis factor‐α (TNF‐α) ELISA Kit	96T
MM‐0181H1	Human Interleukin‐1β(IL‐1β) ELISA Kit	96T
MM‐0055H1	Human Interleukin‐2(IL‐2) ELISA Kit	96T
MM‐0049H1	Human Interleukin‐6(IL‐6) ELISA Kit	96T
MM‐0066H1	Human Interleukin‐10(IL‐10) ELISA Kit	96T
MM‐0839H1	Human calcitonin gene‐related peptide (CGRP) ELISA Kit	96T
MM‐50767H1	Human bradykinin (BK) ELISA Kit	96T
MM‐0761H1	Human 5‐hydroxytryptamine (5‐HT) ELISA Kit	96T
MM‐12905H1	Human lysophosphatidic Acid (LPA) ELISA Kit	96T

Abbreviation: ELISA, enzyme linked immunosorbent assay.
